# Facial geometric feature extraction based emotional expression classification using machine learning algorithms

**DOI:** 10.1371/journal.pone.0247131

**Published:** 2021-02-18

**Authors:** Murugappan M., Mutawa A.

**Affiliations:** 1 Department of Electronics and Communication Engineering, Intelligent Signal Processing (ISP) Research Lab, Kuwait College of Science and Technology (A Private University), Al-Jahra, Kuwait; 2 Computer Engineering Department, College of Engineering and Petroleum, Kuwait University, Kumait, Kuwait; Universiti Malaysia Perlis, MALAYSIA

## Abstract

Emotion plays a significant role in interpersonal communication and also improving social life. In recent years, facial emotion recognition is highly adopted in developing human-computer interfaces (HCI) and humanoid robots. In this work, a triangulation method for extracting a novel set of geometric features is proposed to classify six emotional expressions (sadness, anger, fear, surprise, disgust, and happiness) using computer-generated markers. The subject’s face is recognized by using Haar-like features. A mathematical model has been applied to positions of eight virtual markers in a defined location on the subject’s face in an automated way. Five triangles are formed by manipulating eight markers’ positions as an edge of each triangle. Later, these eight markers are uninterruptedly tracked by Lucas- Kanade optical flow algorithm while subjects’ articulating facial expressions. The movement of the markers during facial expression directly changes the property of each triangle. The area of the triangle (AoT), Inscribed circle circumference (ICC), and the Inscribed circle area of a triangle (ICAT) are extracted as features to classify the facial emotions. These features are used to distinguish six different facial emotions using various types of machine learning algorithms. The inscribed circle area of the triangle (ICAT) feature gives a maximum mean classification rate of 98.17% using a Random Forest (RF) classifier compared to other features and classifiers in distinguishing emotional expressions.

## Introduction

Emotions play a crucial role in interpersonal communication between humans; and humans and machines. It becomes the most popular field of research in the last decade to develop intelligent humanoid robots and human-machine interface (HMI) systems. Facial expressions are among the most potential and straightforward non-verbal communication mediums to create different HMI systems [1–3]. Facial expression recognition methods play a significant role in graphics, 3D-modelling, ache assessment, human-machine interaction, e-learning, clinical analysis, etc. [4]. Though this research field is well-established and has potential methods to efficiently detect facial emotional expressions, there are several challenges in improving the systems’ performance, which effectively works in a real-time environment. Over the last few decades, several algorithms have been proposed by different researchers in detecting facial expressions. Some of the most common facial expression recognition features are more reliable detection, user-friendly approach, cost-effectiveness, and reduced computation complexity [5–8]. Besides, physiological signals, speech, and gestures are also used in HMI system development [8–12]. Universally, emotions are categorized into six classes, such as surprise, anger, sadness, fear, disgust, and happiness. Several kinds of research in the literature attempted to develop intelligent real-time emotion recognition systems [13–16]. Most of the earlier works have utilized a few of the famous international facial expression databases, such as the Japanese Female Facial Expression Database (JAFFE), Cohn-Kanade (CK), Cohn-Kanade extended database (CK+), MMI, etc. for experimenting with their algorithms for facial expression detection [17, 18].

Consequently, the algorithms that analyzed the databases mentioned above were tested over a limited number of samples, utilized still images; mostly worked offline, could not identify macro expressions, and not appropriate for real-time emotional expression detection. Nevertheless, there are few and limited numbers of works focused on facial expression recognition from video sequences. Hence, it becomes indispensable to develop a robust and automated facial emotion recognition system that works online with reduced computational complexity. The present work aims to develop an intelligent and more reliable online facial emotion recognition system using geometrical facial features extracted through the triangulation approach. Ekman and Friesen developed the Facial Action Coding System (FACS) to analyze facial emotional behavior to recognize emotional facial expressions [19, 20]. FACS is designed as a human observer-based system of recognition for detecting minute variations in facial features. This recognition system is fully controllable facial models that manipulate the single actions called Action Units (AUs) or facial action units (FAU). There are 46 action units in FACS and several facial muscles involved for each facial expression. Table 1 illustrates the details of AUs and their locations in facial muscles corresponding to different emotions. These AUs are considered as a reference to identify six different emotional facial expressions. Facial expression recognition algorithms play a significant role in developing intelligent systems over the last few decades [13–16]. Most of the earlier literature works considered different AUs for recognizing emotional expressions [15, 21–24].

**Table 1:** Description of AUs and its locations corresponding to different emotions by Ekman and Friesen [20].

Emotions	Action Units (AU’s)	FACS Name	Muscular Basis
Happiness	AU6	Cheek Raiser	Orbicularis oculi (pars orbitalis)
	AU12	Lip Corner Puller	Zygomaticus major
Sadness	AU14	Dimpler	Buccinator
	AU15	Lip Corner Depressor	Depressor anguli Oris (also known as triangulation)
Surprise	AU12	Lip Corner Puller	Zygomaticus major
	AU5B	Upper Lid Raiser	Levator palpebrae superioris, superior tarsal muscle
	AU26	Jaw Drop	Masseter; relaxed temporalis and internal pterygoid
Fear	AU12	Lip Corner Puller	Zygomaticus major
	AU4	Brow Lowerer	Depressor glabellae, depressor supercilii, corrugator supercilii
	AU5	Upper Lid Raiser	Levator palpebrae superioris, superior tarsal muscle
	AU7	Lid Tightener	Orbicularis oculi (pars palpebralis)
	AU20	Lip Stretcher	Risorius platysma
	AU26	Jaw Drop	Masseter; relaxed temporalis and internal pterygoid
Disgust	AU9	Nose Wrinkler	Levator labii superioris alaeque nasi
	AU15	Lip Corner Depressor	Depressor anguli oris (also known as triangularis)
	AU16	Lower Lip	Depressor labi inferioris
Anger	AU4	Brow Lowerer	Depressor glabellae, depressor supercilii, corrugator supercilii
	AU5	Upper Lid Raiser	Levator palpebrae superioris, superior tarsal muscle
	AU7	Lid Tightener	Orbicularis oculi (pars palpebralis)
	AU23	Lip Tightener	Orbicularis oris

The four main contributions of the present work are, 1. We propose a fully automated facial emotional expression classification system using novel geometric features. 2. We investigate different types of geometric facial features in the triangular form to classify the facial emotional expressions; their significance in distinguishing different emotional expressions are compared. 3. We prove that the representation based on triangle features outperform conventional image-based facial expression recognition with lesser computational complexity. Therefore, our study confirms that triangular elements are highly efficient in discriminating facial emotional expression compared to other types of facial features. 4. We experiment with the proposed methodology extensively in our dataset in facial emotional expression classification. The experimental results of the proposed method outperform the most state-of-the-art facial emotional expression recognition systems. The rest of the paper is organized as follows: Section II presents the related works in facial emotional expression using geometric and appearance-based features. Section III describes the proposed research methodology of emotional expression recognition. Section IV discusses the experimental results and research findings, and the conclusion is presented in Section V.

## Related works

Facial geometric features, appearance-based facial features, and a combination of these two features are mostly utilized in facial expression recognition. In [25] and [26], the researchers have divided the face into many small-size grids, and the features from all these grids are concatenated to identify the facial expressions. However, a trivial misalignment of the face reduces the recognition accuracy due to the extraction of features from inappropriate locations. Region of Interest (ROI) features from facial images and thermal images are used for detecting emotions in child-robot interaction compared to conventional facial AUs based emotion recognition [27]. The computation complexity (both in computational time and memory) of the facial emotion expression recognition system increases proportionally with the number of AUs involved in the design and AUs’ combination. Several approaches have been reported in the literature by analyzing the AUs for facial expression detection. The total number of AUs varied from eight to 250 AUs in the literature and are used for facial emotional expression recognition, and it’s wholly subjective. There is no standard number of AUs have been proposed yet. The total number of AUs and AUs’ locations is based on the application and its requirement [28, 29]. The distance between the AUs is mostly referred to as a standard feature for facial expression recognition. Consequently, researchers have also analyzed the AUs in different forms, such as triangle, net, rectangle, attention map, etc. Here, triangle-based facial expression detection is more prevalent due to lesser computational complexity than conventional marker placement and other geometric feature extraction methods. It also significantly reflects the smallest changes in emotions by changing the properties of a triangle. In [30], the researchers have reported that the performance degrades by the facial expression recognition system by 5% for each year between the test image and training images. The triangular features such as area and perimeter have been used, extracted from eyes, mouth, and nose region using 12 facial action units (FAU), and a maximum face recognition rate of 94% was achieved in the FG-NET aging database. Kuan et.al reported 73.9% of accuracy on emotion recognition using the triangle approach [31]. A total of 48 triangles from 102 facial markers by using the Modified Active Shame Model (MASM) were investigated to extract the features and classified the emotions using Artificial Neural Network (ANN). Chaturvedi and Tripathi also developed a triangular approach based emotion recognition system using a Fuzzy rule-based system [32]. A total of six basic emotions (happiness, anger, fear, surprise, sadness, and disgust) were considered in this research. Fourteen FAUs in the form of reflective stickers are positioned on the subject’s face and connected each marker edges to formulate eight triangles and achieved recognition accuracy of 87.67%. In this work, real time facial emotion recognition is implemented by placing eight virtual markers automatically; and extracting five triangles highlighting perimeter and area of triangle as important features. Ghimire and Lee utilized the angle and position of 52 facial landmark points (FAUs) as geometric features used in the facial emotion expression recognition system [33]. Euclidian distance between each pair of landmarks within a frame and angle are determined. These angle and distance values are subtracted from the respective values in the first frame of the video sequence. Moreover, multiclass AdaBoost is used with dynamic time warping, and SVM operates over the boosted feature vectors. Kamlesh et.al have used a combination of the Active look Model, a geometric approach, and the native Binary Pattern, a look-based approach for facial expression recognition [34]. This hybrid approach is for recognizing emotional facial expressions using 68 different facial feature points. Recently, Hu and Zernike shape-based descriptors model are used facial expressions recognition [35, 36].

For a real-time feeling recognition system, several approaches are projected. A minimal number of AUs are utilized to detect facial expressions [14, 23], despite the varying numbers of AUs used in developing intelligent facial expression recognition system. Delaunay triangulation method is used to connect the 68 facial action units in a subject face to detect seven facial expressions such as happiness, anger, afraid, surprise, sadness, neutral, and disgust. A multiclass support vector machine (SVM) classifier performs well with a maximum mean facial expression recognition rate of 84% based on spatiotemporal features in classifying the emotional expressions [37]. Kartali et al. have reported the results of conventional (SVM, Multi-Layer Perceptron (MLP)) and deep learning methods (Convolutional Neural Network (CNN), Alexnet CNN, Affdex CNN) based facial expression recognition of four emotions (happiness, sadness, anger, and fear) and achieved maximum recognition accuracy of 85.05% using Affdex CNN [38]. A novel vectorized emotion recognition model is proposed to identify three primary emotions: angry, happy, and neutral, using 70 facial vectors and a deep neural network (DNN), and achieved a mean accuracy of 84.33% [39]. In recent literature, the researchers have used spatial and temporal information from input video sequences to classify different facial expressions using CNN, Ensemble Multi-level CNN, and Long Short-term Memory (LSTM) [29, 40–43]. Some of the common issues reported in the earlier works due to the lack of samples or data sets, low accuracy in classifying facial expressions, higher computational complexity (more memory and power required for processing the data), not suitable for real-time applications, and not user-friendly approach (restrictions in using the system for a variety of applications) [44].

This study utilized a simple geometrical feature based on the triangle method to recognize six basic facial expressions (happiness, anger, fear, surprise, sadness, and disgust) using computer-generated markers. The subject’s face is detected by using Haar-like features. The proposed mathematical method places eight automated virtual markers (four markers on the upper face and four makers on the lower face) in a defined location on the subject’s face. The proposed algorithm formulates five different triangles by manipulating eight markers’ positions as an edge of each triangle. Optical Flow Algorithm is used to track each marker movement while facial expression occurs and predicts the future triangle features. The movement of the markers during facial expression directly changes the property of each triangle. Then, the triangle area and perimeter are extracted as the features to classify the facial expressions. These features are statistically validated using one-way analysis of variance (ANOVA) and box plots. Finally, the statistically significant features are mapped into corresponding facial emotion expressions using six nonlinear classifiers, namely, K Nearest Neighbour (KNN), Probabilistic Neural Network (PNN), Random Forest (RT), Decision Tree (DT), Extreme Learning Machine (ELM), and SVM.

## Ethics statement

All the subjects have given their formal written consent and filled up their bio-data form prior voluntarily participating in the experiment. The University ethical committee has suggested that the board approval is not required for this present study.

## Methods and materials

**Fig 1:**
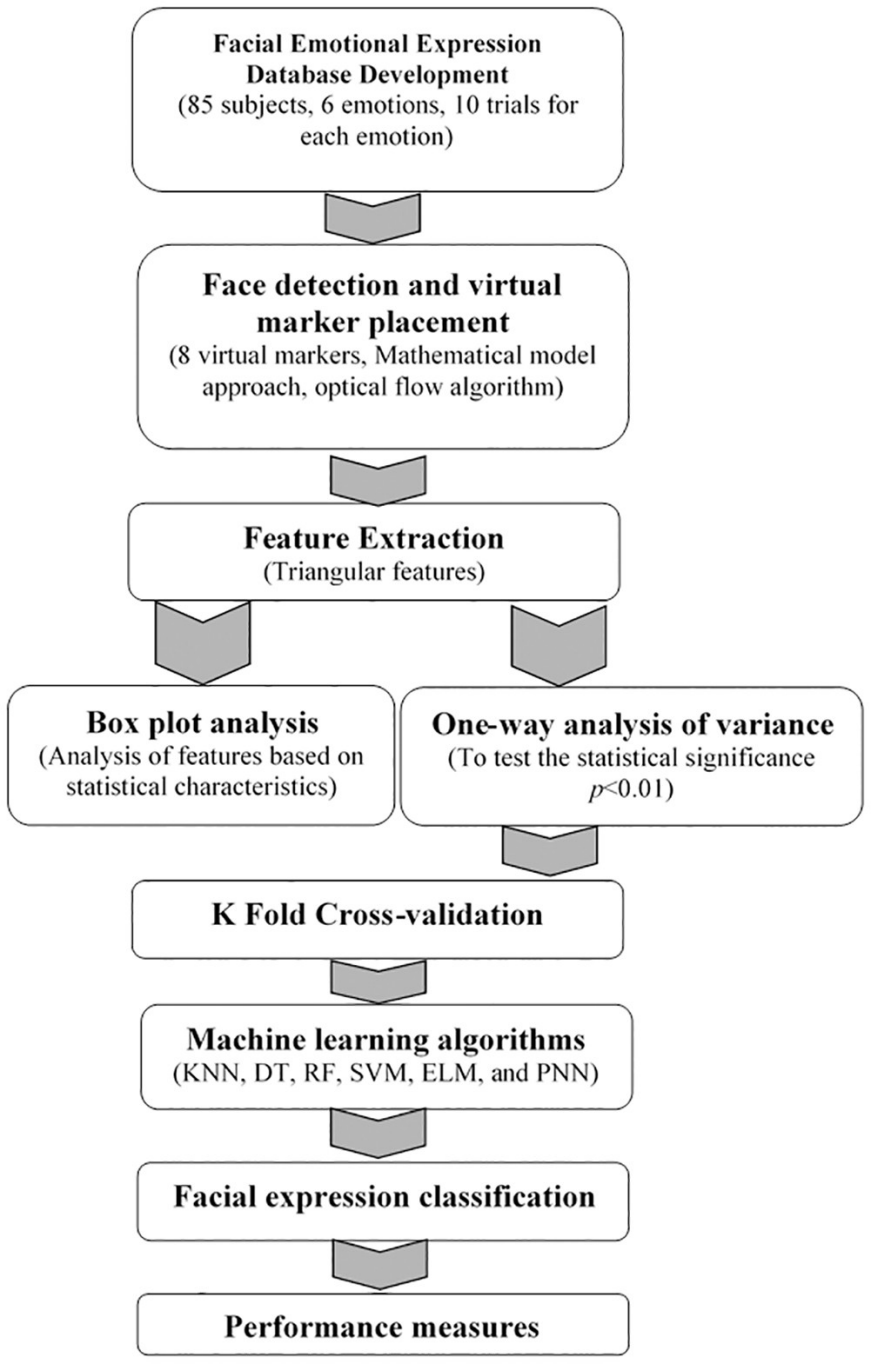
Overview of facial emotion recognition using triangle-based features.

This section describes the various stages of facial emotion expression recognition using computer-generated markers and machine learning algorithms. Fig 1 shows the implementation flow of this present work.

### Facial expression database

This project utilizes the facial expression database developed with multi-ethnic subjects (Malaysian, Indians, Kuwaitis, Syrian, and Chinese) for facial expression recognition. The database consists of facial expression video sequences of six basic emotions (happiness, surprise, anger, fear, disgust, and sadness). Total, 85 undergraduate university students (55 males; 30 female) in the age range of 20—27 years with a mean age of 24.5 years voluntarily participated in this experiment and utilized all these data for developing a facial emotional expression recognition system. All subjects were normal and healthy students with corrected vision and did not have any history related to psychological and muscular disorders. We collected facial images in a controlled environment with 25*°C* ambient room temperature and 50 Lux light intensity using a high-quality built-in HD camera (resolution of 2560 × 1600 at 227 pixels per inch) in the Apple MacBook Pro system. Our preliminary experimental work confirms that the proposed methodology is not affected by any background changes. Hence, the data are collected with different background scenes. All the subjects were asked to seat comfortably in a chair with a distance of 0.95m from the camera, and the camera captured the video sequence at the rate of 30 frames per second (fps). All the subjects have given formal written consent and filled up their bio-data form before starting the experiment.

**Fig 2:**
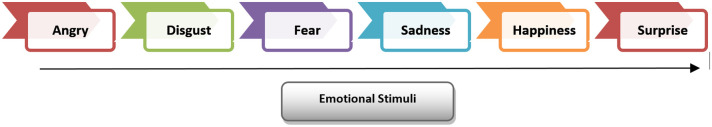
Data acquisition protocol for facial expression recognition database development.

International Affective Picture System (IAPS) images of six different emotions (sadness, happiness, fear, anger, surprise, and disgust) with higher intensity (Valence –Arousal) are selected based on the heuristic approach. They used to formulate the computerized protocol to guide the subject to express their emotions autonomously. The flow of emotional stimuli of one trial is shown in Fig 2. Before starting the stimuli, a set of instructions will be shown to the subjects for 10 seconds, followed by a natural scene image of 10 seconds to neutralize the subject’s feelings. This database is collected with ten trials, and a 10-sec break is given to the subjects between the trials. Each trial has six emotions, and each emotion has six images. Images of a given emotion last for 6 sec. The computing system continuously records the marker positions in (X,Y) coordinates and saves them as comma-separated values (CSV) format for subsequent processing stages.

### Face and eye detection

**Fig 3:**
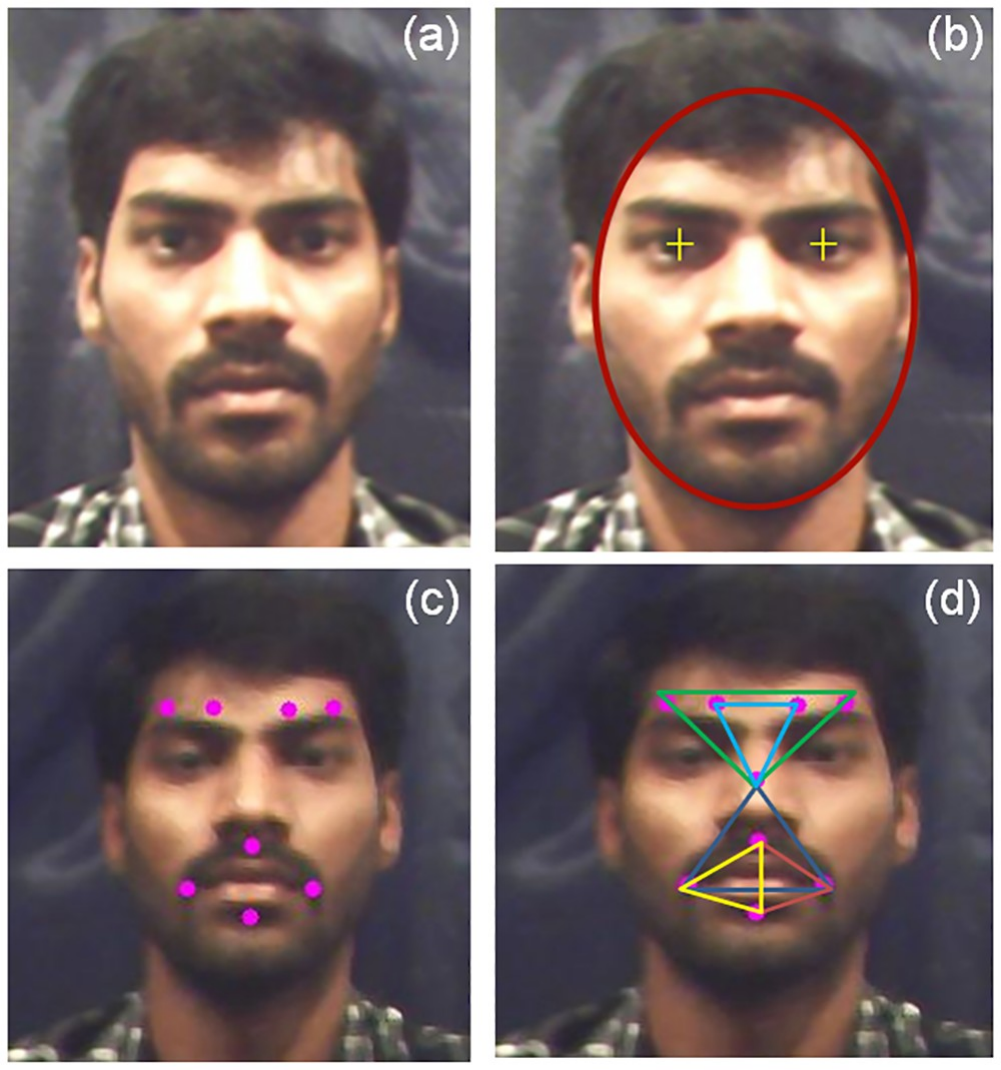
Automatic face detection and computer-generated marker placement; (a) subject facial image, (b) eye and face detection, (c) subject’s face with eight computer-generated markers, (d) triangle model.

Automated face recognition should accurately detect the subject’s face with lesser computational complexity (computational time and memory). The eyes, mouth, and nose served as primary reference points to identify users’ faces in the scene [45]. Though different face detection methods were reported in recent years [14, 45], the Viola and Jones face detection method is mostly referred to in earlier works for real-time facial emotion recognition than other face detection methods [4, 16, 46]. Viola and Jones have utilized Haar-like features in the detection of the face, eyes, nose, and mouth [46]. Haar-like features were used to compute pixel contrast (white and black) between adjacent rectangular groups using lines, edges, and center-surround features instead of using the image’s original pixel values in face detection. Thereby, this method required lesser computation time and memory for face detection than other methods [46]. Recently, AdaBoost cascade classifier employed in Haar-like features to detect human face in the scene efficiently [48]. OpenCV library is utilized in the present work to capture the image sequences from the webcam. The captured image sequences are converted from color image to greyscale image before implementing the face detection method. Consequently, the subject’s face and eyes in the video were detected using Haar-like features. The face detection using this proposed method is faster than other methods in the literature (computation time: 0.067 sec) [47]. Finally, the algorithm proposed in our earlier work [47] formulates an ellipse around the face, positioned “+” markers on both eyes of the subject’s to ease the process of computer-generated marker placement [48, 49]. A sample subject after face and eye detection is shown in Fig 3(a) and 3(b), respectively.

### Automated marker placement and triangle approach

**Fig 4:**
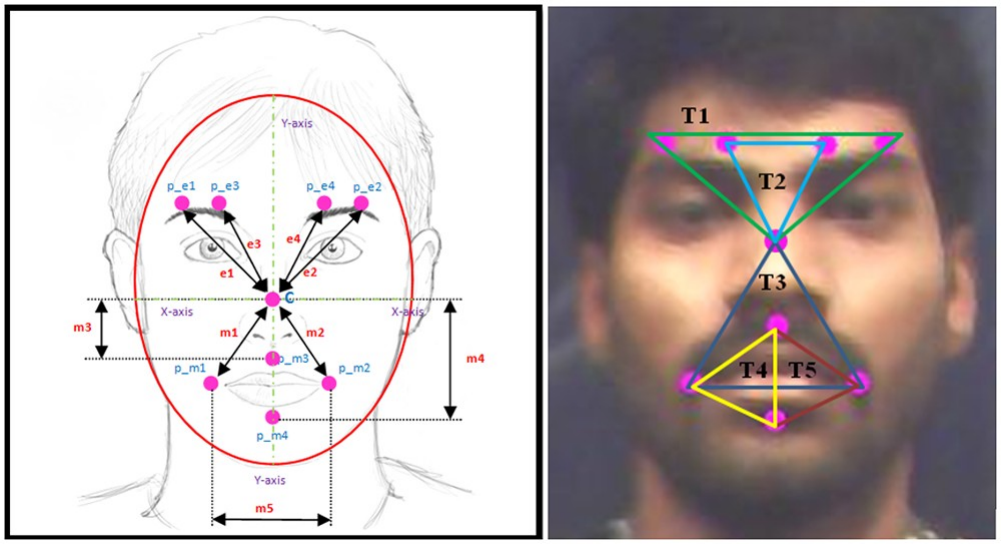
A geometrical model of an automated marker and proposed triangle with the label.

We proposed a mathematical model for placing eight virtual markers on a defined location in the subject’s face [49]. Descriptions of the computer-generated marker placement algorithm for emotional facial expressions can be found in [49]. After the marker placement, each marker’s edges are used to formulate five triangles for facial emotional expression detection. We have investigated a maximum of 42 different triangles by heuristic approach, and finally, five triangles are selected based on higher emotional expression rate. Fig 3 shows the face detection, automated marker placement and proposed triangle method. Table 2 describes the correspondent triangle formulated through facial features (markers) and Fig 4 shows the geometrical model of marker placement. Here, p_e1 refers to the first marker in the left eye, p_e2 refers to the second marker in the right eye, p_e3 refers to the second marker in the left eye, p_e4 refers to the first marker in the right eye, C center marker, p_m1 refers to the marker in the left side of the mouth, p_m2 refers to the marker in the right side of the mouth, p_m3 refers to the marker in the top of the mouth, and p_m4 refers to the marker at the bottom of the mouth.

**Table 2:** Facial features (markers) and associated triangles.

Triangles	Facial features (markers)
Triangle 1 (T1)	p_e1, p_e2 & C (centre)
Triangle 2 (T2)	p_e3, p_e4 & C (centre)
Triangle 3 (T3)	p_m1, p_m2 & C (centre)
Triangle 4 (T4)	p_m1, p_m3 & p_m4
Triangle 5 (T5)	p_m2, p_m3 & p_m4

### Lucas-Kanade optical flow algorithm

There are several methods used in real-time emotional facial expression detection, such as local phase quantization [50], pyramid histogram of gradient [51], the FACS [52], local binary patterns [53], and the optical flow algorithm (OFA) [54, 55]. The OFA has been widely used to identify changes in the AUs for facial emotion detection in the real-time environment [55, 56]. In general, the OFA aims to identify and calculate an object’s motion pattern in a visual scene caused by the relative motion between an observer and the background [57]. The OFA can be implemented in any of the following methods: phase correlation, block-based methods, discrete optimization, and differential methods [58]. Phase correlation estimates the relative translative offset using a fast frequency-domain approach between two similar images [59]. Block-based methods are used to maximize the normalized cross-correlation or minimize the sum of absolute or squared differences of two identical images in a scene. Differential methods are based on partial derivatives of images and include the Lucas–Kanade, Horn–Schunck, Buxton–Buxton, Black–Jepson, and general variation methods [60, 61]. Optical flow aims to predict the future pixel position and its velocity using the values in a current frame [62, 63]. The OFA implementation of this work has three assumptions: brightness consistency, temporal persistence, and spatial coherence [63]. In this present work, the Lucas-Kanade OFA in Open CV is implemented to track facial markers thereby the changes of triangle features can be easily investigated for facial emotion recognition.

### Triangular feature extraction

In the present work, the distance between each marker and the Center of the image (C) is calculated using Pythagoras’ Theorem [32] (Eq 1). Since every marker has its X-Y coordinates (the centre point is (Xc, Yc), the distance of each marker concerning Centre (C) of the facial image can compute by using Pythagoras’ Theorem. The unknown ‘i’ and ‘j’ in the Eq 1 represent two points concerned to find the correspondent distance. So, the X-Y coordinates of three facial features (markers) are used to formulate one triangle (shown in Table 2). From Pythagoras’ Theorem,

$${\left(\text{Hypotenuse}\right)}^{2}={\left(X_{i}-X_{j}\right)}^{2}+{\left(Y_{i}-Y_{j}\right)}^{2}$$ (1)

Therefore, the formula for distance of each marker’s is calculated by Eq (2):

$$\begin{array}{c} \text{Distance}=\sqrt{{\left(X_{i}-X_{j}\right)}^{2}+{\left(Y_{i}-Y_{j}\right)}^{2}} \end{array}$$ (2)

**Fig 5:**
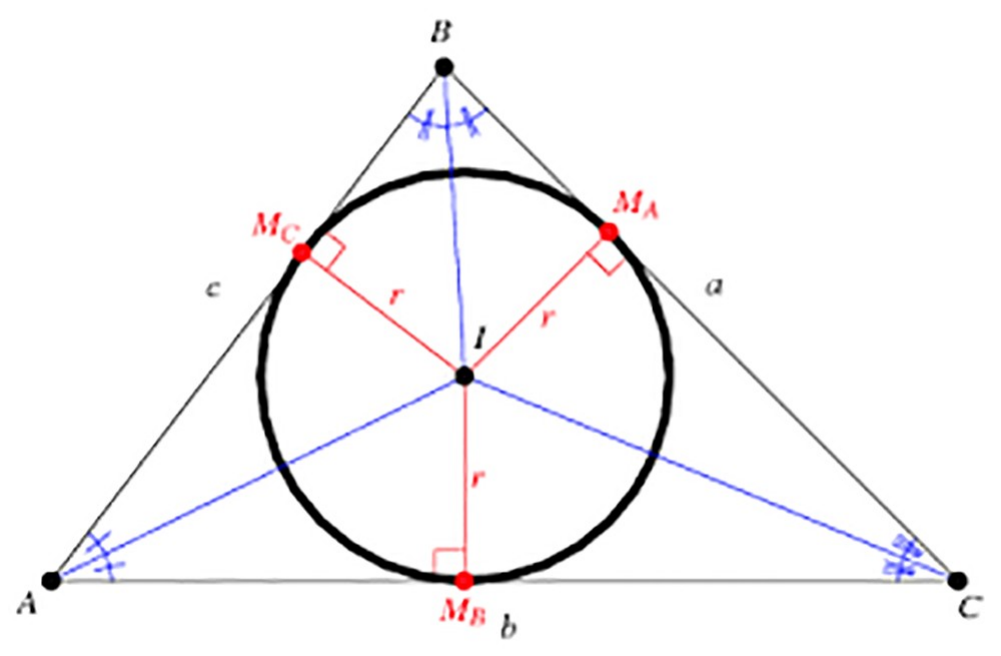
Inscribed circle in a triangle.

After the formulation of five triangles using eight virtual markers, an inscribed circle (Fig 5) is created inside the triangle to compute three simple statistical features, namely, Inscribed circle area of a triangle (ICAT), Inscribed Circle Circumference (ICC), and Area of a triangle (AoT). Fig 5 shows the inscribed circle of a triangle. Here, A, B, and C refer to the virtual markers. The intersection of three edges creates a center point of the circle (l). The distance between l to M_A or M_B or M_C refers to the radius of the circle (r). Here, A, B, C refers three markers used to formulate the triangle. These features are highly significant in identifying the more acceptable changes in triangle property during emotional facial expressions than other statistical features [31, 32]. Finally, these features are used for classifying different facial expressions using machine learning algorithms. The mathematical equations for deriving these features are.

#### Area of a Triangle (AoT)

The area of a triangle can be used as a simple statistical feature to detect emotions. In this work, Heron’s formula is used to calculate the area of a triangle [25], and the formula for the area of triangle computation is given in Eq (3). Here, the unknown parameters such as D1, D2, and D3 have considered three distances from the triangle.

$$\begin{array}{c} \text{Area}(\text{Triangle})\;=\sqrt{S(S-D1)(S-D2)(S-D3)} \end{array}$$ (3)

where S is the mean value of three edge distances and defined by,

$$\begin{array}{c} =\frac{\left(D1+D2+D3\right)}{2} \end{array}$$ (4)

#### Inscribed Circle Circumference (ICC)

This is used to measure the circumference of the Inscribed circle [34].

$$\begin{array}{c} \text{Circumference}\;=2\pi r \end{array}$$ (5)

where the radius (r) of the triangle is determined by,

$$\begin{array}{c} r=2*\frac{\text{Area}(\text{Triangle})}{\text{Perimeter}} \end{array}$$ (6)

The perimeter of the triangle can be computed as,

Perimeter=D1+D2+D3 (7)

#### Inscribed Circle Area of a Triangle (ICAT)

The area of the Inscribed circle is computed as,

$$\begin{array}{c} A\left(\text{Inner}\;\text{Circle}\right)=\pi r^{2} \end{array}$$ (8)

where r is the radius of the inner circle.

### Statistical analysis of features

In this work, box plot analysis and ANOVA are used to investigate the significance of the triangular features in facial emotion expression recognition.

#### One-way analysis of variance

The value of significance level p<0.01 is kept as a threshold in identifying the features’ statistical significance in distinguishing emotions. In this work, only the significant features are used for facial expression classification. SPSS toolbox is used for performing ANOVA.

#### Box plot analysis

Box plot analysis analyzes the characteristics of features in emotional expression classification. Initially, the min-max normalization method is applied to the extracted features to transform the data into the range of 0—1. Later, the basic statistical features such as minimum, maximum, standard deviation, 25% quartile, 50% quartile, 75% quartile are used to devise the box plot. The box plot gives information about the characteristics of features in distinguishing emotions.

### Facial emotion classification

In this work, six different machine learning algorithms such as KNN, ELM, PNN, RF, DT, and SVM are used to classify facial emotion.

#### K nearest neighbour

KNN is a simple nonlinear classifier used in several applications, including epilepsy detection, driver drowsiness detection, emotion recognition, seizure detection, and many other problems. KNN is a non-probabilistic learning algorithm used to classify unknown test data based on the majority of similar data among the k-nearest neighbors closest to test/anonymous data. Different distance measures can measure the distance between the test data and each training data, such as Manhattan, Euclidean, Minkowski, and Chebyshev. In this work, the above four distance measures are used to distinguish facial emotional expressions, and the mean Accuracy of each distance measure is reported in Section IV. Besides, the value of k-nearest neighbor has been varied from 1 and 10 with unit step increment. The value of k-neighbour, which gives the highest accuracy, is reported in Section IV.

#### Decision Tree (DT)

Decision Tree (DT) is a supervised machine learning algorithm, and it principally works on the concept of statistical prediction and modeling. This classifier can understand the definitive decision-making knowledge from the training data. This classifier is mostly used in data modeling, data mining, machine learning, and decision-making applications.

#### Probabilistic neural network (PNN)

Probabilistic neural network (PNN) is one of the most popular machine learning algorithms used for classification and pattern recognition applications. In PNN, the value of standard deviation (*σ*) is varied with a step value of 0.01 in the ranges of 0.01 to 0.9. The value of (*σ*) which produces a maximum mean classification rate is reported in Section IV.

#### Random forest

Random forest classifier is ensemble learning method used for classification, regression, and pattern recognition applications. The basic principle of this classifier is built on constructing the decision during training time based on the characteristics of the data and gives the output based on the characteristics of testing data, which matches training. The performance of the classifier is based on the number of trees used for classification. In this work, the number of trees in the RF algorithm has been varied heuristically between 20 and 500. The value of tree at which the classifier gives the maximum mean emotion classification rate is reported in Section IV.

#### Extreme learning machine

Extreme learning machine (ELM) play a significant role in pattern recognition, and pattern classification applications. This employs a feed-forward neural network having only one hidden layer unlike conventional neural network architecture. Due to the simple and layered architecture, this classification computationally fast compared to other machine learning algorithms. Two kernel functions are used in the output layer such as Radial Basis Function (RBF) and Multi-Layer Perceptron (MLP) to classify the emotions. We investigated four different activation functions (tanh, sigmoid, Gaussian, and hardlim) in the MLP kernel for evaluation of comparative performance. The grid search method is used to determine the optimal value of RBF width in the ranges of 0.01 to 0.1 with a step value of 0.01 and the hidden neurons of 1000—2500 with a step value of 100.

#### Support vector machine

The support vector machine is a nonlinear and supervised learning method used for several biomedical and image processing applications. In general, SVM is developed for two-class problems, and the provision of kernel functions extend the application of SVM in multiclass problems. In this work, we used a Radial Basis Function as a kernel for emotion classification. The classifier’s performance depends on the value of cost function (c) and kernel factor (*γ*). This work utilized the grid-search approach for finding the optimal value of c from 2^−14^ to 2^+15^ and *γ* from 2^−14^ to 2^+8^ for getting a higher classification rate.

## Experimental results and discussion

This section discusses the experimental results of different triangular features in facial emotional expression classification using machine learning algorithms.

### One-way analysis of variance and box plot analysis

**Fig 6:**
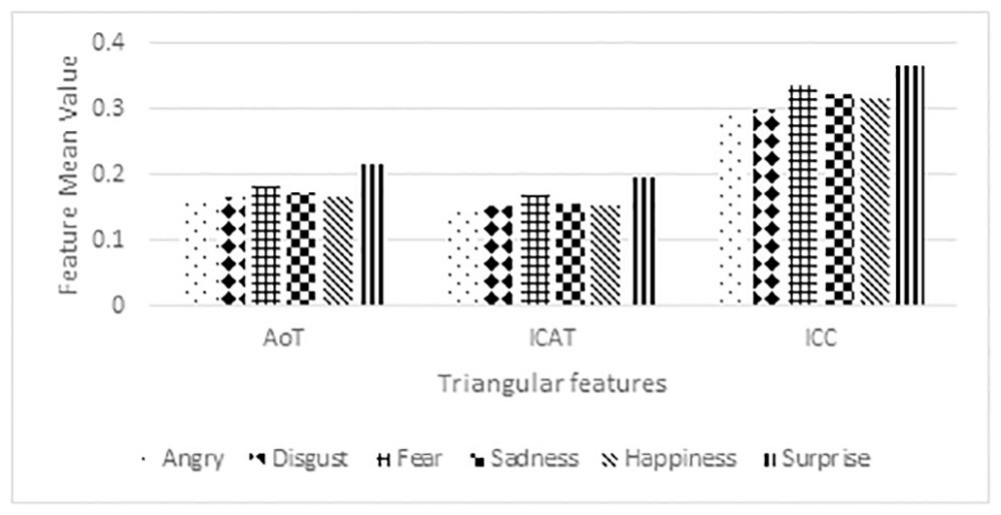
Distribution of the mean value of triangular features in emotion classification.

The mean values of three different emotional features over six emotional expressions are shown in Fig 6. The inscribed circle circumference of the triangle and the triangle’s inscribed area have the highest and lowest normalized feature mean values, respectively. The area of the triangle feature plays an intermediate role in terms of the normalized feature values. Compared to the circumference feature, the area of the triangle and area of the inscribed circle show different patterns among the emotions in the subjects. In specific, the intense emotional perception is reflected in surprise (positive) and followed by fear (negative) emotion. The feature distribution also conveys that the negative emotions such as disgust, anger, and sadness are not expressed accurately by the subjects through these emotions felt strongly by the subjects.

**Fig 7:**
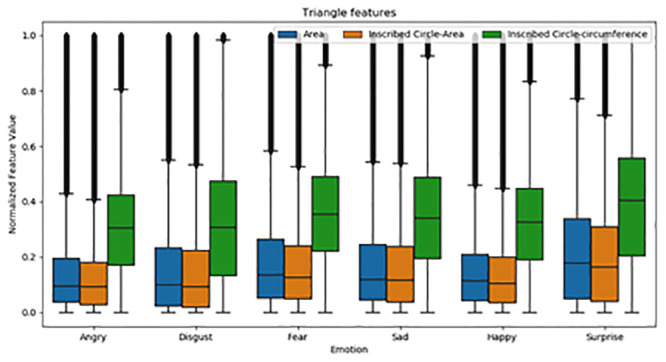
Distribution of the mean value of triangular features in emotion classification.

Table 3 shows the F value and p value of ANOVA analysis for three different features in emotional expression classification. The proposed features have a highly significant value (p < 0) and achieved a high F value, which indicates the efficacy of the features in distinguishing six emotional expressions by having higher variance between the emotional features and smaller variance within the emotional features. The inscribed circle circumference has a high F value, followed by the inscribed circle area and area of the triangle. The results of ANOVA analysis confirm that the proposed features have higher significance in distinguishing six different emotions. Besides, the box plot (in Fig 7) clearly shows that all three statistical features have a different mean value and offer a more considerable variance between the emotions. Among the three features, the ICAT feature has a higher normalized feature value distribution between the emotions and effectively distinguishes the other two features’ emotions.

**Table 3:** Significant test results of statistical features using ANOVA.

S.No	Feature	F Value	p-Value
1	Inscribed circle circumference	1894.34392	11.00 × 10^−14^
2	Inscribed circle area of triangle	712.7374704	19.40 × 10^−9^
3	Area of Triangle	625.8698817	12.20 × 10^−18^

#### Facial emotion classification

In this work, six different machine learning algorithms are used for facial emotional expression classification. In general, the performance of machine learning algorithms in emotional expression classification mainly depends on the appropriate selection of network hyper parameters. There are several methods proposed in the literature for hyper parameter selection, and mostly, the heuristic approach is reported in the literature for finding the optimal value. Table 4 illustrates the performance of facial emotion expression classification using the area of triangle feature employing six different machine learning algorithms with the optimal hyper parameter values used in emotional expression classification. It is interesting to note that most of the classifiers gave a maximum mean classification rate (> 95%) besides ELM. Though the ELM classifier is a computationally fast and single feed-forward neural network, it could not classify the emotions with higher accuracy than other classifiers. RF classifier outperforms the other classifier by giving a maximum mean classification rate of 97.97%. There is no significant difference in classification performance noted in KNN with different distance measures.

**Table 4:** Facial emotional expression classification using the area of triangle (AoT) features.

Classifier	Kernel function / Distance measure	Hyper parameters	Mean Accuracy (in %)
SVM	RBF	C = 2^+13^, gamma = 2^+6^	95.37
PNN	Default	sigma = 0.08	96.17
Decision tree	Default		96.17
Random Forest	No of trees = 300		97.97
KNN	Euclidean	K = 6	96.17
	Manhattan	K = 6	96.50
	Minkowski	K = 8	96.33
	Chebyshev	K = 16	96.17
ELM	RBF-Gaussian	RBF_width = 0.05	91.00
	MLP-tanh	No of Hidden neurons = 3000	93.67
	MLP-Gaussian		93.00
	MLP-Sigmoid		92.50
	MLP-Hardlim		93.50

**Fig 8:**
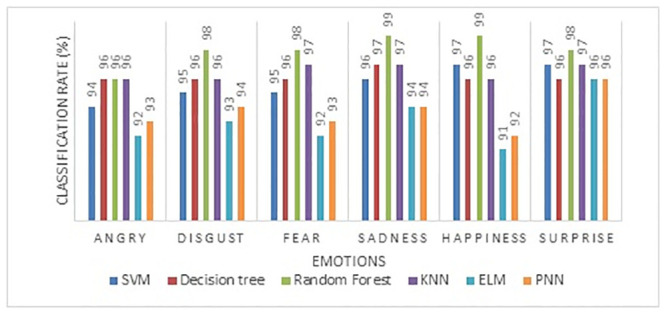
Individual emotion classification rate of area of triangle feature (AoT).

The individual emotion classification rate of emotions of six different machine learning algorithms is shown in Fig 8. The two extremes of emotions, such as happiness and sadness, have been effectively identified with a maximum recognition rate using the triangle feature area. Also, the negative emotions such as disgust, anger, and fear efficiently are classified by the triangle feature area using RF classifier compared to other classifiers. SVM classifier achieved a lower classification rate in all six types of emotions compared to other classifiers.

The performance of the inscribed circle area of triangle (ICAT) feature attained a maximum mean accuracy of 98.17% in detecting all six types of emotions using an RF classifier compared to the area of triangle feature (Table 5). This indicates the smallest changes in the triangle area could be captured by the ICAT feature in emotional expression classification. RF classifier is one of the simple classifiers that requires limited hyperparameters tuning compared to other nonlinear classifiers. The changes in the circle have effectively captured the smallest changes in the facial expressions.

**Table 5:** Facial emotional expression classification using the inscribed circle area of triangle (ICAT) features.

Classifier	Kernel function / Distance measure	Hyper parameters	Mean Accuracy (in %)
SVM	RBF	C = 2^+14^, gamma = 2^+5^	96.55
PNN	Default	sigma = 0.08	96.17
Decision tree	Default		96.33
Random Forest	No of trees = 500		98.17
KNN	Euclidean	K = 8	96.33
	Manhattan	K = 6	96.50
	Minkowski	K = 14	96.33
	Chebyshev	K = 10	96.17
ELM	RBF-Gaussian	RBF_width = 0.05	92.33
	MLP-tanh	No of Hidden neurons = 3000	93.33
	MLP-Gaussian		93.17
	MLP-Sigmoid		92.50
	MLP-Hardlim		93.50

**Fig 9:**
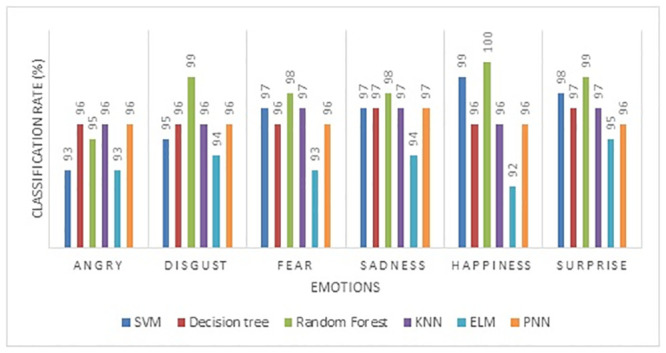
Individual emotion classification rate of inscribed circle area of triangle feature.

The individual classification rate of the ICAT feature gives a maximum mean accuracy of 100% in happiness and 99% in surprise and disgust emotion (Fig 9). Compared to the AoT feature, ICAT gives a higher classification rate in detecting all six types of emotions. Though the emotional expressions of negative emotions such as sadness, anger, fear, and disgust could not be expressed sturdily by a subject, the proposed feature (ICAT) effectively captures even smaller or subtle changes in the marker positions. The performance of emotion classification using the Inscribed Circle Circumference (ICC) feature is given in Table 6. The maximum mean classification rate is 97.47% using the RF classifier, and consequently, the ELM classifier gives the lowest mean classification rate of 94.17% compared to other classifiers. The KNN classifier’s accuracy with Manhattan distance achieved a maximum accuracy of 97% in facial expression recognition.

**Table 6:** Facial emotional expression classification using inscribed circle circumference features.

Classifier	Kernel function / Distance measure	Hyper parameters	Mean Accuracy (in %)
SVM	RBF	C = 2^+15^, gamma = 2^+7^	96.00
PNN	Default	sigma = 0.01	96.33
Decision tree	Default		96.50
Random Forest	No of trees = 550		97.47
KNN	Euclidean	K = 5	96.33
	Manhattan	K = 2	97.00
	Minkowski	K = 6	96.33
	Chebyshev	K = 12	96.33
ELM	RBF-Gaussian	RBF_width = 0.05	93.83
	MLP-tanh	No of Hidden neurons = 3000	94.17
	MLP-Gaussian		94.00
	MLP-Sigmoid		93.83
	MLP-Hardlim		93.83

**Fig 10:**
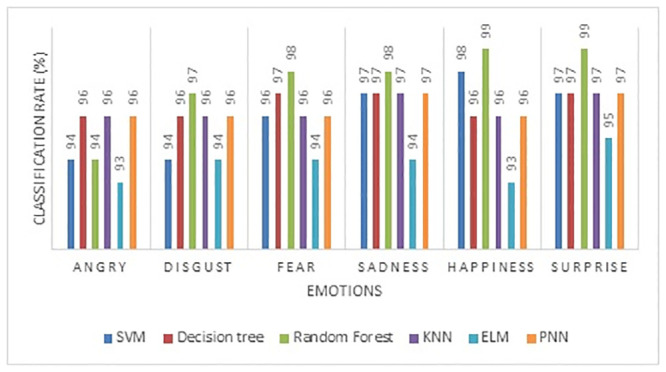
Individual emotion classification rate of inscribed circle circumference feature.

**Fig 11:**
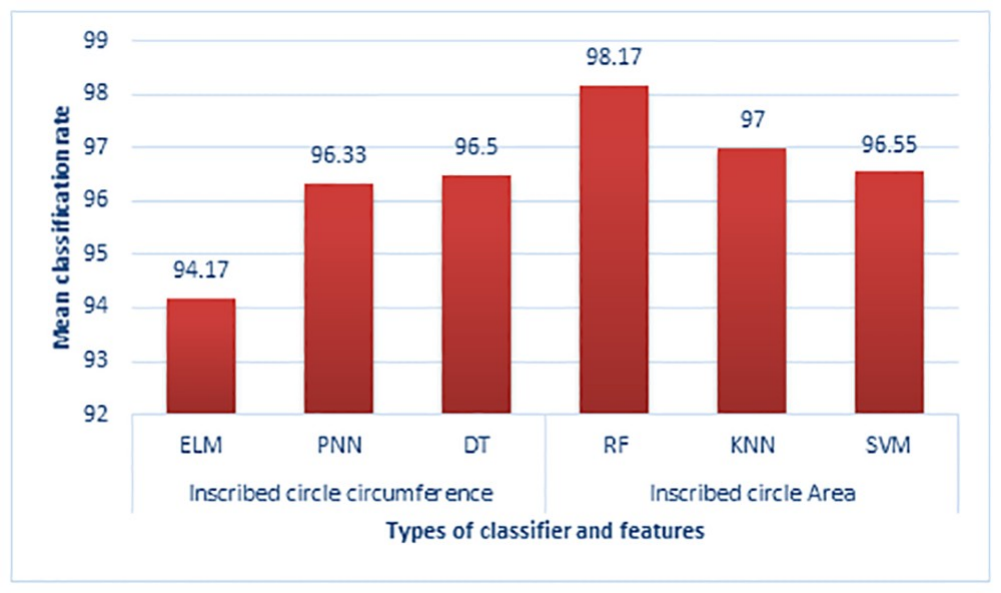
Performance comparison of classifiers in facial emotional expression recognition.

The individual emotion classification rate of the ICC feature is shown in Fig 10. The positive emotions (happiness and surprise) achieved a maximum recognition rate compared to negative emotions (disgust, sadness, anger, and fear) using the RF classifier. Among the negative emotions, the angry emotion achieved a lower recognition rate of 94% in all the classifiers. The proposed ICC feature commendably distinguishes the negative and positive emotions using the RF classifier. Among the three features presented in this paper, the ICAT achieved a maximum accuracy of 98.17% through the RF classifier compared to the other features and classifiers (shown in Fig 11).

#### Performance comparison

The accuracy of facial emotional expression classification using three proposed features (AoT, ICAT, and ICC) is compared with the earlier works which utilize the same database. Some of the common issues in the previous works are: (a) most of the previous works used the international standard databases of static images with a limited number of samples (b) utilized fixed or reflective markers to track the facial expressions (c) most of the experiments are performed in a constrained environment with a limited number of emotional expressions, (d) utilized more extensive set of FAUs to detect facial expressions, and (e) variety of algorithms are proposed for recognizing facial expressions in an offline environment. To address the issues In [49] and [64], we have utilized a smaller number of samples for facial expression recognition and analyzed a simple distance measure to distinguish facial expressions. However, in the present work, we analyzed the facial emotional expressions of 85 subjects from five different nationalities (India, Kuwait, Malaysia, China, and Syria). We achieved a maximum mean accuracy of 98.17% with lesser computation time and reduced memory requirement. Though the accuracy of our earlier method using deep neural network (LSTM) achieved 99.80%, the methodology requires more computational power (graphical processing units (GPUs)) and computation time than the proposed work. This present work’s complete experiment has been performed in an Intel I7 processor with 8 GB RAM in a Windows operating system using Python and Open CV software. Table 7 shows the comparison of classifier rate from previous work to current work. In this present work, we proposed three new features using the triangulation method (ICC, AoT, and ICAT) compared to the distance measure in classifying emotional expressions. The proposed feature (Inscribed Circle Area of Triangle) achieved a maximum mean accuracy of 98.17% with a limited computation time and computation power.

**Table 7:** Comparing recognition rates with earlier works.

Ref No	No of virtual markers	No of Subjects	Features	Classifiers	Max mean accuracy (in %)
[49]	8	30	Distance	PNN	96.94
[64]	8	55	Distance	PNN	92.00
[29]	10	85	Distance	LSTM	99.80
Present work	8	85	Inscribed Circle Area of triangle	RF	98.17

Though the present work is efficient and straightforward in classifying emotional expressions using a limited number of AUs and triangulation methods, few limitations still exist, (a) the present work does not analyze the marker movement during face rotation and action. (b) the proposed face emotional expression algorithm could be compared with an international standard database for facial expressions in a video sequence (c) proposed features could be used to classify the emotional expression using different types of deep neural networks (DNN) (d) the robustness and performance of the proposed algorithm could be tested with the subjects with different age groups.

## Conclusion

Researchers are interested in developing a reliable and straightforward facial expression recognition system that effectively classifies different categorical emotions in real-time applications. This present work proposes facial geometry features based triangulation method to classify the facial emotional expressions in real-time. Eight virtual markers referred to as Action Units (AUs) were placed on the subject’s face on a defined location, and a combination of three AUs was used to formulate five triangles. Three new features, namely the Inscribed Circle Area of Triangle (ICAT), Area of Triangle (AoT), Inscribed Circle Circumference (ICC) are extracted from five triangles. These features are cross-validated using the fivefold-cross validation method and classified using six machine learning algorithms. The random forest (RF) classifier gives the maximum accuracy of 98.17% in classifying six emotions (happiness, anger, sadness, fear, disgust, and surprise) using the ICAT feature. All the three proposed features give a maximum accuracy (>90%) in classifying facial emotional expressions. The proposed method utilized a larger number of samples (85 subjects and six emotions). It analyzed triangular features to classify the emotional expressions using machine learning algorithms and achieved a higher classification rate than earlier works.
